# Supplementary material for: Effectiveness of Influenza Vaccination and Early Antiviral Treatment in Reducing Pneumonia Risk in Severe Influenza Cases
Source: Vaccines (Basel). 2024 Feb 7;12(2):173. doi: 10.3390/vaccines12020173 (PMC10891622; doi:10.3390/vaccines12020173)
Supplement: Supplementary file 1 [file vaccines-12-00173-s001.zip › vaccines-2819718-supplementary.pdf]

Table S1. Patients 18-64 years with severe influenza with and without pneumonia during the 2010/2011-2019/2020 flu seasons in Catalonia (Spain).

	With pneumonia (N=1314)	Without pneumonia (N=572)	OR (95%CI)	aOR (95% CI)
Influenza vaccination				
Yes	133 (10.3%)	79 (14.3%)	0.69 (0.51-0.93)	0.95 (0.68-1.34)
No	1156 (89.7%)	473 (85.7%)	Ref.	Ref.
Sex				
Female	555 (42.2%)	255 (44.6%)	0.91 (0.75-1.11)	
Male	759 (57.8%)	317 (55.4%)	Ref.	
ICU admission				
Yes	496 (37.7%)	179 (31.3%)	1.33 (1.08-1.64)	
No	818 (62.3%)	393 (68.7%)	Ref.	
Death				
Yes	118 (9.0%)	35 (6.1%)	1.51 (1.02-2.24)	1.70 (1.08-2.68)
No	1196 (91.0%)	537 (93.9%)	Ref.	Ref.
≥1 comorbidities				
Yes	794 (60.4%)	438 (76.6%)	0.47 (0.37-0.58)	
No	520 (39.6%)	134 (23.4%)	Ref.	
COPD				
Yes	179 (13.7%)	152 (27.0%)	0.43 (0.34-0.55)	0.44 (0.34-0.58)
No	1132 (86.3%)	412 (73.0%)	Ref.	Ref.
Obesity				
Yes	116 (8.9%)	90 (16.0%)	0.51 (0.38-0.69)	0.53 (0.38-0.74)
No	1189 (91.1%)	473 (84.0%)	Ref.	Ref.
Diabetes				
Yes	207 (15.8%)	105 (18.6%)	0.82 (0.64-1.07)	
No	1099 (84.2%)	460 (81.4%)	Ref.	
CKD				
Yes	97 (7.4%)	45 (7.9%)	0.93 (0.64-1.34)	
No	1213 (92.6%)	523 (92.1%)	Ref.	
Immunodeficiency				
Yes	265 (20.3%)	111 (19.7%)	1.04 (0.81-1.33)	
No	1043 (79.7%)	453 (80.3%)	Ref.	
CVD				
Yes	195 (14.9%)	93 (16.5%)	0.88 (0.68-1.16)	
No	1116 (85.1%)	471 (83.5%)	Ref.	
CLD				
Yes	109 (8.3%)	46 (8.2%)	1.02 (0.71-1.46)	
No	1199 (91.7%)	516 (91.8%)	Ref.	
Antiviral treatment				
Yes	1247 (95.0%)	529 (92.8%)	1.46 (0.98-2.19)	
No	66 (5.0%)	41 (7.2%)	Ref.	
Antiviral treatment				
≤48h before symptom onset	330 (26.0%)	221 (40.3%)	Ref.	Ref.
>48h after symptom onset	873 (68.8%)	286 (52.2%)	2.04 (1.65-2.54)	1.91 (1.51-2.41)
No	66 (5.2%)	41 (7.5%)	1.08 (0.70-1.65)	0.84 (0.53-1.35)
Hospital stay, days				
0-14	906 (69.1%)	412 (72.2%)	0.86 (0.69-1.07)	0.84 (0.66-1.08)
>14	406 (30.9%)	159 (27.8%)	Ref.	Ref.

ARDS				
Yes	461 (35.7%)	315 (55.5%)	0.44 (0.36-0.54)	
No	830 (64.3%)	253 (44.5%)	Ref.	
Multiorgan failure				
Yes	126 (9.8%)	33 (5.8%)	1.76 (1.18-2.61)	
No	1158 (90.2%)	533 (94.2%)	Ref.	
Influenza virus type				
A	1071 (81.5%)	486 (85.0%)	Ref.	
B	242 (18.4%)	84 (14.7%)	1.31 (0.99-1.71)	
C	1 (0.1%)	2 (0.3%)	0.23 (0.02-2.51)	
Influenza season				
2010-11	97 (7.4%)	31 (5.4%)	3.04 (1.86-4.96)	2.07 (1.20-3.54)
2011-12	32 (2.4%)	7 (1.2%)	4.44 (1.87-10.52)	3.65 (1.47-9.08)
2012-13	45 (3.4%)	15 (2.6%)	2.91 (1.52-5.56)	2.19 (1.08-4.45)
2013-14	122 (9.3%)	46 (8.0%)	2.57 (1.66-3.99)	2.22 (1.35-3.64)
2014-15	126 (9.6%)	22 (3.8%)	5.56 (3.27-9.45)	4.02 (2.27-7.12)
2015-16	209 (15.9%)	51 (8.9%)	3.98 (2.63-6.01)	3.20 (2.01-5.08)
2016-17	72 (5.5%)	38 (6.6%)	1.84 (1.14-2.98)	1.62 (0.93-2.82)
2017-18	280 (21.3%)	112 (19.6%)	2.43 (1.70-3.46)	1.91 (1.28-2.86)
2018-19	230 (17.5%)	152 (26.6%)	1.47 (1.04-2.07)	1.10 (0.74-1.64)
2019-20	101 (7.7%)	98 (17.1%)	Ref.	Ref.

Abbreviations: ARDS: acute respiratory distress syndrome. CI: confidence interval. CKD: chronic kidney disease. CLD: chronic liver disease. COPD: chronic obstructive pulmonary disease. CVD: cardiovascular disease. ICU: intensive care unit. OR: odds ratio.

Supplementary Table S2. Patients 65-74 years with severe influenza with and without pneumonia during the 2010/2011-2019/2020 flu seasons in Catalonia (Spain).

	With pneumonia (N=643)	Without pneumonia (N=409)	OR (95%CI)	aOR (95% CI)
Influenza vaccination				
Yes	218 (34.2%)	152 (38.3%)	0.84 (0.65-1.09)	0.94 (0.71-1.25)
No	419 (65.8%)	245 (61.7%)	Ref.	Ref.
Sex				
Female	241 (37.5%)	143 (35.0%)	1.12 (0.86-1.44)	
Male	402 (62.5%)	266 (65.0%)	Ref.	
ICU admission				
Yes	171 (26.6%)	78 (19.1%)	1.54 (1.14-2.08)	1.42 (1.01-2.01)
No	472 (73.4%)	331 (80.9%)	Ref.	Ref.
Death				
Yes	102 (15.9%)	43 (10.5%)	1.60 (1.10-2.35)	1.41 (0.92-2.17)
No	541 (84.1%)	366 (89.5%)	Ref.	Ref.
≥1 comorbidities				
Yes	533 (82.9%)	363 (88.8%)	0.61 (0.42-0.89)	
No	110 (17.1%)	46 (11.2%)	Ref.	
COPD				
Yes	210 (32.7%)	176 (43.0%)	0.64 (0.50-0.83)	0.63 (0.48-0.84)
No	433 (67.3%)	233 (57.0%)	Ref.	Ref.
Obesity				
Yes	61 (9.6%)	56 (13.7%)	0.67 (0.45-0.98)	0.75 (0.57-0.99)
No	574 (90.4%)	352 (86.3%)	Ref.	Ref.
Diabetes				

Yes	214 (33.3%)	154 (37.9%)	0.82 (0.63-1.06)	
No	429 (66.7%)	252 (62.1%)	Ref.	
CKD				
Yes	99 (15.4%)	69 (16.9%)	0.89 (0.64-1.25)	
No	544 (84.6%)	339 (83.1%)	Ref.	
Immunodeficiency				
Yes	166 (25.8%)	85 (20.8%)	1.32 (0.98-1.78)	
No	477 (74.2%)	323 (79.2%)	Ref.	
CVD				
Yes	234 (36.4%)	158 (38.7%)	0.91 (0.70-1.17)	
No	408 (63.6%)	250 (61.3%)	Ref.	
CLD				
Yes	41 (6.4%)	24 (5.9%)	1.09 (0.65-1.84)	
No	599 (93.6%)	383 (94.1%)	Ref.	
Antiviral treatment				
Yes	611 (95.0%)	390 (95.4%)	0.93 (0.52-1.66)	
No	32 (5.0%)	19 (4.6%)	Ref.	
Antiviral treatment				
≤48h before symptom onset	216 (34.4%)	158 (40.3%)	Ref.	
>48h after symptom onset	380 (60.5%)	215 (54.8%)	1.29 (0.99-1.68)	
No	32 (5.1%)	19 (4.8%)	1.23 (0.67-2.25)	
Length of hospital stay, days				
0-14	438 (68.2%)	287 (70.2%)	0.91 (0.70-1.20)	
>14	204 (31.8%)	122 (29.8%)	Ref.	
ARDS				
Yes	207 (33.1%)	253 (62.9%)	0.29 (0.22-0.38)	
No	419 (66.9%)	149 (37.1%)	Ref.	
Multiorgan failure				
Yes	68 (11.0%)	28 (7.0%)	1.64 (1.04-2.60)	
No	553 (89.0%)	374 (93.0%)	Ref.	
Influenza virus type				
A	483 (75.1%)	336 (82.2%)	Ref.	
B	160 (24.9%)	73 (17.8%)	1.52 (1.12-2.08)	
C	-	-		
Influenza season				
2010-11	18 (2.8%)	2 (0.5%)	10.76 (2.38-48.60)	6.54 (1.41-30.42)
2011-12	18 (2.8%)	7 (1.7%)	3.08 (1.19-7.94)	2.86 (1.08-7.63)
2012-13	15 (2.3%)	6 (1.5%)	2.99 (1.08-8.27)	2.21 (0.78-6.38)
2013-14	31 (4.8%)	23 (5.6%)	1.61 (0.84-3.10)	1.49 (0.72-3.10)
2014-15	53 (8.2%)	19 (4.6%)	3.34 (1.76-6.34)	2.87 (1.47-5.60)
2015-16	103 (16.0%)	32 (7.8%)	3.85 (2.24-6.63)	3.34 (1.88-5.92)
2016-17	68 (10.6%)	50 (12.2%)	1.63 (0.97-2.74)	1.49 (0.84-2.63)
2017-18	170 (26.4%)	96 (23.5%)	2.12 (1.35-3.32)	1.99 (1.23-3.23)
2018-19	116 (18.0%)	113 (27.6%)	1.23 (0.78-1.93)	1.12 (0.68-1.83)
2019-20	51 (7.9%)	61 (14.9%)	Ref.	Ref.

Abbreviations: ARDS: acute respiratory distress syndrome. CI: confidence interval. CKD: chronic kidney disease . CLD: chronic liver disease. COPD: chronic obstructive pulmonary disease. CVD: cardiovascular disease. ICU: intensive care unit. OR: odds ratio.

Supplementary Table S3. Patients ≥75 years with severe influenza with and without pneumonia during the 2010/2011-2019/2020 flu seasons in Catalonia (Spain).

	With pneumonia (N=1267)	Without pneumonia (N=875)	OR (95%CI)	aOR (95% CI)
Influenza vaccination				
Yes	609 (48.9%)	441 (51.5%)	0.90 (0.76-1.07)	0.84 (0.70-1.02)
No	637 (51.1%)	415 (48.5%)	Ref.	Ref.
Sex				
Female	669 (52.8%)	413 (47.2%)	0.80 (0.67-0.95)	0.71 (0.58-0.87)
Male	598 (47.2%)	462 (52.8%)	Ref.	Ref.
ICU admission				
Yes	164 (12.9%)	91 (10.4%)	1.28 (0.98-1.68)	1.28 (0.94-1.75)
No	1103 (87.1%)	784 (89.6%)	Ref.	Ref.
Death				
Yes	231 (18.2%)	157 (17.9%)	1.02 (0.82-1.28)	
No	1036 (81.8%)	718 (82.1%)	Ref.	
≥1 comorbidities				
Yes	1079 (85.2%)	741 (84.7%)	1.04 (0.82-1.32)	
No	188 (14.8%)	134 (15.3%)	Ref.	
COPD				
Yes	336 (26.6%)	261 (29.9%)	0.85 (0.70-1.03)	0.71 (0.57-0.89)
No	926 (73.4%)	612 (70.1%)	Ref.	Ref.
Obesity				
Yes	66 (5.4%)	63 (7.3%)	0.72 (0.51-1.03)	0.65 (0.44-0.96)
No	1164 (94.6%)	803 (92.7%)	Ref.	Ref.
Diabetes				
Yes	403 (32.1%)	256 (29.6%)	1.12 (0.93-1.35)	
No	854 (67.9%)	609 (70.4%)	Ref.	
CKD				
Yes	314 (24.9%)	231 (26.4%)	0.92 (0.76-1.12)	
No	949 (75.1%)	643 (73.6%)	Ref.	
Immunodeficiency				
Yes	172 (13.7%)	109 (12.6%)	1.10 (0.85-1.42)	
No	1088 (86.3%)	759 (87.4%)	Ref.	
CVD				
Yes	622 (49.2%)	407 (46.7%)	1.11 (0.93-1.32)	
No	641 (50.8%)	465 (53.3%)	Ref.	
CLD				
Yes	47 (3.7%)	27 (3.1%)	1.21 (0.75-1.95)	
No	1214 (96.3%)	842 (96.9%)	Ref.	
Antiviral treatment				
Yes	1136 (90.1%)	822 (94.2%)	0.56 (0.40-0.79)	
No	125 (9.9%)	51 (5.8%)	Ref.	
Antiviral treatment				
≤48h before symptom onset	381 (31.4%)	387 (46.5%)	Ref.	Ref.
>48h after symptom onset	708 (58.3%)	394 (47.4%)	1.82 (1.51-2.20)	1.73 (1.41-2.12)
No	125 (10.3%)	51 (6.1%)	2.49 (1.75-3.55)	2.05 (1.40-2.99)
Length of hospital stay, days				
0-14	980 (77.4%)	630 (72.0%)	1.33 (1.09-1.62)	1.33 (1.06-1.67)
>14	286 (22.6%)	245 (28.0%)	Ref.	Ref.
ARDS				
Yes	361 (29.4%)	573 (65.9%)	0.22 (0.18-0.26)	
No	866 (70.6%)	297 (34.1%)	Ref.	
Multiorgan failure				

Yes	93 (7.6%)	84 (9.7%)	0.77 (0.56-1.05)	
No	1128 (92.4%)	783 (90.3%)	Ref.	
Influenza virus type				
A	964 (76.1%)	688 (78.6%)	Ref.	
B	303 (23.9%)	186 (21.3%)	1.16 (0.94-1.43)	
C	0 (0%)	1 (0.1%)	-	
Influenza season				
2010-11	12 (0.9%)	8 (0.9%)	1.77 (0.69-4.53)	1.64 (0.60-4.51)
2011-12	44 (3.5%)	14 (1.6%)	3.70 (1.90-7.22)	2.76 (1.36-5.60)
2012-13	27 (2.1%)	10 (1.1%)	3.18 (1.46-6.95)	2.30 (0.94-5.63)
2013-14	69 (5.4%)	52 (5.9%)	1.56 (0.99-2.48)	1.29 (0.77-2.18)
2014-15	151 (11.9%)	52 (5.9%)	3.42 (2.23-5.26)	3.29 (2.05-5.28)
2015-16	120 (9.5%)	31 (3.5%)	4.56 (2.79-7.45)	4.40 (2.56-7.55)
2016-17	216 (17.0%)	135 (15.4%)	1.89 (1.31-2.71)	1.53 (1.01-2.32)
2017-18	335 (26.4%)	248 (28.3%)	1.59 (1.14-2.22)	1.41 (0.96-2.06)
2018-19	209 (16.5%)	226 (25.8%)	1.09 (0.77-1.54)	0.95 (0.64-1.40)
2019-20	84 (6.6%)	99 (11.3%)	Ref.	Ref.

Abbreviations: ARDS: acute respiratory distress syndrome. CI: confidence interval. CKD: chronic kidney disease . CLD: chronic liver disease. COPD: chronic obstructive pulmonary disease. CVD: cardiovascular disease. ICU: intensive care unit. OR: odds ratio.
